# Grafting: a potential method to reveal the differential accumulation mechanism of secondary metabolites

**DOI:** 10.1093/hr/uhac050

**Published:** 2022-02-28

**Authors:** Ding Dong, Ya-Na Shi, Zong-Min Mou, Sui-Yun Chen, Da-Ke Zhao

**Affiliations:** 1 Biocontrol Engineering Research Center of Plant Disease and Pest, Yunnan University, Kunming, 650504, China; 2 Biocontrol Engineering Research Center of Crop Disease and Pest, Yunnan University, Kunming, 650504, China; 3 School of Life Science, Yunnan University, Kunming, 650204, China; 4 Institute of Medicinal Plants, Yunnan Academy of Agricultural Sciences, Kunming, 650000, China; 5 School of Ecology and Environmental Science, Yunnan University, Kunming, 650504, China

## Abstract

Plant secondary metabolites make a great contribution to the agricultural and pharmaceutical industries. Their accumulation is determined by the integrated transport of target compounds and their biosynthesis-related RNA, protein, or DNA. However, it is hard to track the movement of these biomolecules *in vivo*. Grafting may be an ideal method to solve this problem. The differences in genetic and metabolic backgrounds between rootstock and scion, coupled with multiple omics approaches and other molecular tools, make it feasible to determine the movement of target compounds, RNAs, proteins, and DNAs. In this review, we will introduce methods of using the grafting technique, together with molecular biological tools, to reveal the differential accumulation mechanism of plant secondary metabolites at different levels. Details of the case of the transport of one diterpene alkaloid, fuziline, will be further illustrated to clarify how the specific accumulation model is shaped with the help of grafting and multiple molecular biological tools.

## The accumulation of secondary metabolites is regulated by multiple factors

**Figure 1:**
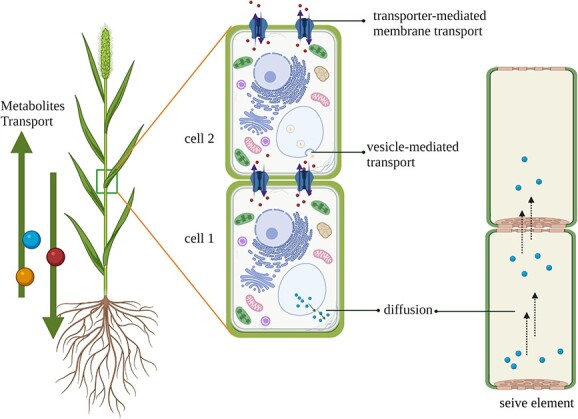
Three main transport modes of metabolites. Diffusion, vesicle-mediated transport, and transporter-mediated membrane transport. Different-colored balls represent different metabolites.

Plant secondary metabolites are usually defense compounds responding to various biotic and abiotic stresses [1–3]. They are also the main sources of one-third of clinical drugs and their use is still increasing [4, 5]. Secondary metabolites customarily accumulate in specific tissues in plants. In the tissues where they accumulate these metabolites are mainly dependent on the transport of intermediates or final products, as well as the RNAs and proteins responsible for the biosynthesis of target secondary metabolites [6]. Previously, Knowledge of the transport mechanisms of these metabolites mostly focused on the direct transport of compounds, primarily including diffusion, vesicle-mediated transport, and transporter-mediated membrane transport [7] (Fig. 1). Regarding these metabolites, the transport of root-biosynthesized nicotine to leaves resulted in the abundant accumulation of this alkaloid in tobacco leaves [8]. Berberine also exhibited a similar transport model, in which the compound biosynthesized in goldthread roots would translocate to the rhizome and accumulate there [9].

Until now, little information has been available regarding the RNAs and proteins responsible for biosynthesis due to the lack of attention paid to secondary metabolites. It has been demonstrated that extensive movability of RNAs and proteins occurs in model plants like *Arabidopsis* and tobacco [10–14], indicating the possibility of transfer of these RNAs and proteins, which are closely connected with secondary metabolite biosynthesis. Therefore, the accumulation of particular target compounds could be potentially reflected by the transport of the metabolites, RNA, proteins, and other factors associated with their biosynthesis.

Grafting has been proved to be able to change the accumulation of secondary metabolites [15–17], and presents an emerging method to investigate transport mechanisms at the different levels mentioned above.

## Grafting is a potential method to study the transport and accumulation mechanisms of secondary metabolites

Grafting is a traditional horticulture technique used for improving crop qualities [18–21]. Particularly, grafted plants showed differential metabolite composition and accumulation compared with non-grafted plants [15–17, 22]. Elevated levels of salicylic acid, benzoic acid, vanillin, lignin, and polyamines accumulated in grafted peppers compared with ungrafted ones [23]. Greater accumulation of lycopene was also reported in the fruits of grafted watermelon [24]. Together with the enhancement of accumulation of particular secondary compounds, grafting can also be used to determine their transport directions. A typical case is *Arabidopsis* mutant grafting. Exploring the long-distance movement of metabolites is the initial motivation for *Arabidopsis* grafting [25, 26]. Transport directions of several metabolites, like phytochelatins [27] and GA12 (gibberellic acid 12) [28, 29], were successfully determined by *Arabidopsis* mutant grafting. By grafting different *Arabidopsis* genotypes or even different species, such as the wild type and a mutant genotype lacking the particular metabolite or pathway, the appearance of one target metabolite in the mutant would signify its mobility [25, 26]. With the rapid development of molecular technology and multiomics analysis, specific metabolites [30–32], as well as RNAs [11, 17, 33, 34], proteins [35–37], and even DNA [37–40], were found to be transferred between scions and rootstocks [41]. Their broad transport implies that the differential accumulation of secondary metabolites caused by grafting may not be restricted to the direct transport of compounds but also involves RNAs, proteins, and DNA.

In a nutshell, grafting can change the composition and accumulation of metabolites in sampled plants, and therefore provides an ideal experimental model for studying the transport and accumulation mechanisms of secondary metabolites.

**Figure 2:**
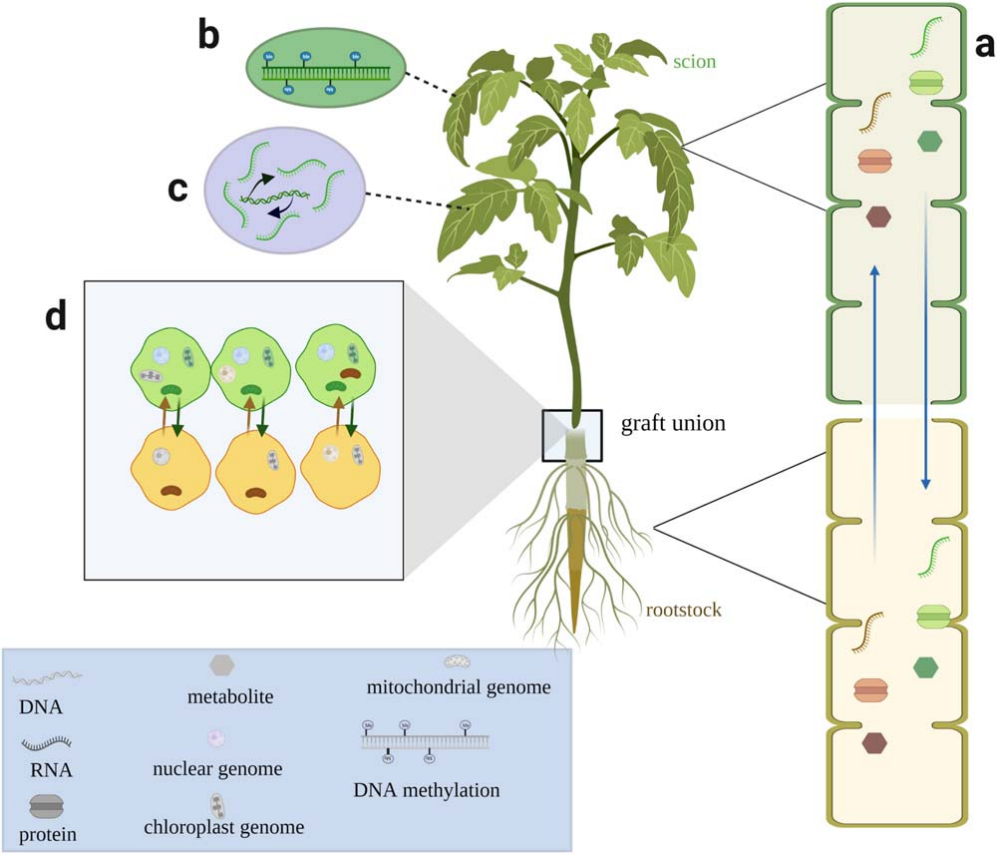
Schematic view of differential accumulation mechanism of secondary metabolites in a grafted plant. Green icons represent molecules from the scion; brown and purple represent molecules from the rootstock. **a** Transfer of metabolites, RNA, and proteins. **b** DNA methylation. **c** Transcriptional enhancement. **d** DNA translocation at graft union.

## Grafting leads to direct compound transport

Long-distance chemical transport in grafted plants contributes significantly to the differential accumulation of specific secondary metabolites [42–44] (Fig. 2a). A typical example is nicotine. This compound was principally considered to be produced in tobacco leaves, with the observation that leaves accumulated a greater content of nicotine than either roots or stems. However, many subsequent investigations were irreconcilable with this view. First, nicotine in tobacco leaves appeared to bear no constant relationship to the size, growth, or any other morphogenetic or physiological characteristic of the leaves [45]. Secondly, most tobacco leaves kept a low content of nicotine in the detached aerial parts of the plants when tobacco leaves and shoots in various developmental stages were removed from the roots [45–47]. These results suggested that nicotine may not be produced in the aerial portions at all but produced largely or perhaps solely in roots. This hypothesis was further confirmed by mutual grafting tests of tomato and tobacco [8, 48, 49]. As expected, the nicotine content increased by more than 50 times in tomato scion when tobacco was used as stock; however, it decreased by almost 90% in tobacco scion when grafted to a tomato stock [8, 49]. This grafting study indicated that nicotine was primarily synthesized underground and then transported over a long distance, eventually accumulating in leaves.

The case of nicotine transport provides a general idea of how to use grafting to study the transport mechanism of secondary metabolites. But there is a practical limitation in that there is a strict need for a significantly different content of target compounds in scion and stock, or the absence of the target compound in one of them. This places a complicated requirement on the choice of which scions and stocks to combine, and may even make grafting not possible for some constitutive compounds. Mutant grafting or isotope labeling provides an alternative approach to providing a fundamental solution to this problem. Taking mutant grafting as an example, 8-methylsulfinlyloctylglucosinolate (8MSO) is a constitutive secondary metabolite exclusively found in the Capparales [50]. Wild-type *Arabidopsis* and 8MSO-free mutant grafting proved that the 8MSO that accumulated principally in seeds was mostly transported from scion leaves, together with a small portion from the rosette leaves [50].

Isotope labeling, coupled with mutant grafting, could be utilized to deeply explore the transport and accumulation of secondary metabolites. Gene mutation of the biosynthesis pathway of the target compound produces a target-chemical-deficient plant, but the presence of intermediates in the metabolic pathway downstream of the mutated gene could result in the emergence of the target compound. In other words, the new accumulation of target metabolites in the mutant caused by grafting may be the result of direct transport not only of the metabolites themselves, but also of their metabolic intermediates. Combined with mutant grafting, isotope labeling is a potential solution to this problem. For instance, reciprocal grafting with different abscisic acid (ABA)-deficient mutants and wild-type genotypes successfully confirmed the transport of ABA in aerial organs to roots [51]. The transfer of ABA itself was further confirmed by spraying isotope-labeled [^2^H_6_]ABA on grafted tomato leaves and detecting the labeled ABA in the roots [51]. Grafting is also used to investigate the transporters associated with secondary metabolites through a series of experiments, including mutant grafting and isotope labeling. A long-distance transport mechanism of cytokinins in *Arabidopsis* mediated by cytokinin transporter AtABCG14 was successfully illustrated recently via the proposed approaches [52].

## Grafting induces DNA short-distance transport and DNA methylation

DNAs could move from one plant species to another by grafting [53] (Fig. 2d). These mobile DNAs include not only the chloroplast genome and mitochondrial genome but also the nuclear genome [37, 38, 40, 54, 55] (Fig. 2d). The exchange of DNA through the graft junction was firstly observed by grafting tobacco with different antibiotic resistance and fluorescence in the stock chloroplast genome and the scion nuclear genome [39]. A series of follow-up experiments successfully proved the mobility of chloroplast [38], mitochondrial [55], and nuclear genomes [37, 54]. Regarding secondary metabolites biosynthesized, accumulated, or regulated in chloroplasts [56, 57] or mitochondria [58, 59], if the chloroplast genome and mitochondrial genome were transported over a long distance, it could theoretically anticipated to be detected in the remote graft partner, but evidence for this has not been obtained yet. Grafting does not appear to facilitate the long-distance transport of DNA between scion and stock, because the sexual reproduction of grafted plants still follows a similar phenotype to the scion rather than the rootstock. In fact, DNA transport is specifically localized in the contact zone between scion and rootstock [60]. This was verified by the appearance of the two entire nuclear genomes from both scion and stock in the allopolyploid species generated by culturing cells at graft junctions [37]. For grafted plants, the short-distance transport of genomes around the graft union, therefore, partially determines the transport and accumulation of secondary metabolites.

Grafting can alter the methylation of DNA as well (Fig. 2b). For example, grafted rubber (*Hevea brasiliensis*) scions exhibited epigenetic changes after grafting to genetically distinct rootstocks [61]. As another case, when the Valencia (VO) citrus scion variety was grafted on the two rootstocks Rangpur lime (RL) and Sunki Maravilha (SM), the two combinations presented polymorphic alterations of epigenetic marks for this crop [62]. Additionally, there was a significant increase in global DNA methylation in cucumber and melon scions after heterografting onto pumpkin rootstocks [63]. These results showed the universality of DNA methylation induced by grafting.

DNA methylation induced by grafting seems to be heritable, due to the fact that the sexual progenies of scions displayed methylation patterns similar to those of heterografting-derived scions [17, 64]. The transport of 15 variant MSAP (methylation-sensitive amplified polymorphism) loci that occurred in tomato scions or eggplant scions to their respective self-pollinated progenies was detected; moreover, at least 11 of out these 15 loci showed a high level of inheritance (83.3–100%) [64]. What is worth mentioning is that the heritability of DNA methylation is reversible over generations. In grafting between *Brassica juncea* and *Brassica oleracea*, DNA methylation in grafting-selfing generation 1 exhibited 5.29–6.59% methylation changes compared with parental plants, and 31.58% of these changes were stably transmitted to grafting-selfing generation 5, but the remainder reverted to the original status over generations [65]. Despite the lack of a direct relationship between graft-induced DNA methylation and secondary metabolites, evidence for DNA methylation affecting secondary metabolism in non-grafted plants has been found [66], suggesting the possibility of grafting-induced DNA methylation that directly or indirectly enables regulation of the synthesis, transport, or accumulation of secondary metabolites.

Since current studies document that DNA can be transported over a only short distance by the graft union, we tentatively consider that DNA transport is not the pivotal element for the accumulation of secondary metabolites. The heritability of DNA methylation and its potential regulation for secondary metabolism implies that the transport and accumulation of secondary metabolites in grafted plants may be mainly regulated by epigenetic modification rather than DNA transport.

## Grafting induces transcription enhancement and RNA transport

In general, the accumulation of secondary metabolites is positively correlated with the expression of their biosynthetic genes. Grafting has been reported to stimulate the expression of these specific structural genes, thereby increasing the synthesis and accumulation of related products [22, 67]. Graft-induced transcriptional enhancement (Fig. 2c) is caused by two factors: grafting itself and the interaction between scion and rootstock [68–70].

The transcriptional enhancement induced by grafting itself was evidenced by self-grafting [68]. A 2.5-fold increase in transcription of argonaute gene 1 was detected in self-grafted tomato compared with its non-grafted counterpart [68], implying the ability of the graft itself to overexpress specific genes. Although the mechanism of transcriptional enhancement induced by grafting itself is still unknown, researchers tend to regard grafting as a stimulus or stress and regard the transcriptional enhancement as a result of the response to this stimulus/stress [48]. It is also considered one of the reasons why self-grafting is often used as a control in heterografting surveys.

Interaction between heterogenous rootstock and scion promoted transcription enhancement. When a grapevine grafting used the ‘Gaglioppo’ variety as scion and grafted it onto 13 different rootstocks, 5 of the 13 grafted grapevine leaves kept a high variability in gene expression, especially significant modulation of transcripts linked to primary and secondary metabolism (e.g. through the upregulation of ~40 genes coding for stilbene synthases) [67]. In contrast to self-grafting (watermelon/watermelon), grafting watermelon onto bottle gourd and squash rootstocks, respectively, induced the differential expression of 787 and 3485 genes associated with primary and secondary metabolism, hormone signaling, and transcription factors [71].

Aside from transcriptional enhancement, grafting could facilitate RNA mobility through the graft union as well [72] (Fig. 2a). The graft-mediated long-distance-transported RNA principally includes mRNAs and small RNAs [73]. Three stock-derived mRNAs responsible for auxin signal transduction were transferred from melon stock to pumpkin scion [74]. *GIBBERELLIC ACID INSENSITIVE* (*GAI*) mRNA, as a transcriptional regulator of gibberellic acid response genes, was demonstrated to be transported via the graft union and functioned at the shoot apex of the scion [75–77]. Along with mRNA, several specific small RNAs, like microRNA and siRNA, could also travel a long distance through the graft union. Grafting microRNA-miR399-overexpressing *Arabidopsis* scion onto the wild-type rootstock of *Arabidopsis* led to the accumulation of miR399 in wild-type roots [78, 79]. Graft transmission of miR172 from overexpressing scion to wild-type potato stock was observed [80]. Furthermore, endogenous siRNA could move through the graft union and function in the recipient cells as well [81], and it has been expected that mobile small RNAs potentially function as something like epigenetic modification messages in the plant body [81].

Despite the accumulated documentation of RNA transport in grafted plants, it has rarely been shown whether long-distance-transportable RNA is directly involved in secondary metabolite synthesis. The synthesis and accumulation of secondary metabolites are regulated by a couple of factors, including hormones [82–84] and transcriptional regulatory factors [85–87]. Some mobile RNAs have been proved to participate in the regulation of hormones like gibberellic acid [75–77] or as transcriptional regulators [88] to regulate plant metabolism. This suggests that the contribution of mobile RNA to the accumulation of secondary metabolites is to some extent achieved through indirect regulation or a series of cascade processes.

## Grafting promotes the increased accumulation and transport of proteins

Grafting induces the accumulation of specific proteins. The accumulated protein responsible for secondary metabolites, intriguingly, has been successfully verified in graft plants [89]. A homograft of pecan exhibited a dynamic proteome change in which ~49 proteins in >2-fold expression difference were associated with multiple metabolic processes compared to un-grafted pecan, including secondary metabolism [90]. Additionally, five proteins related to flavonoid biosynthesis were observed by the comparative proteomic analysis of graft unions in hickory [16].

**Figure 3:**
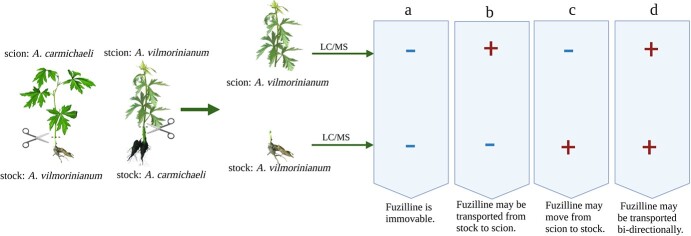
Grafting *Aconitum vilmorinianum* and *A. carmichaeli* onto each other determine the transport direction of fuziline. Fuziline is specifically accumulated in *A. carmichaeli* rather than in *A. vilmorinianum.* The minus sign (−) represents the lack of fuziline, while + represents the existence of fuziline in samples. **a** Detection of fuziline in neither scion nor stock of *A. vilmorinianum* suggests the immovability of fuziline. **b** Observation of fuziline in *A. vilmorinianum* scion but not in *A. vilmorinianum* stock indicates that fuziline may be transported from the underground part to the aboveground part. **c** Observation of fuziline in *A. vilmorinianum* stock but not in *A. vilmorinianum* scion indicates fuziline may be transported from the aboveground part to the underground part. **d** If fuziline is detected in both *A. vilmorinianum* scion and stock, fuziline may be transported bidirectionally.

Typically, the content of a protein is positively correlated to the expression of its coding mRNA in most cases, particularly when the target traits have appeared. As mentioned above, grafting stimulates and promotes a large accumulation of mRNAs in specific tissues. Not surprisingly, grafting would eventually elevate their translated proteins [16, 90, 91]. Together with mRNA translation, the proteins could be directly transported a long distance from scion to stock or vice versa, independently of their coding mRNAs (Fig. 2a). Investigations have shown that pear polygalacturonase-inhibiting proteins (pPGIP) were successfully detected in scions but without the appearance of PGIP-encoding RNA when grafting wild-type tomato and grapevine onto their corresponding pPGIP-expressing rootstocks [92]. Moreover, RT–PCR and northern blotting demonstrated that protein products rather than mRNA transcripts were translocated across graft junctions when two cucurbit structural P-proteins, PP1 and PP2, were examined in intergeneric grafts [93]. Under some circumstances, proteins and their coding mRNAs could be transferred together from donors to recipients. It has been widely confirmed that FLOWERING LOCUS T (FT) protein, a systemic florigenic signal, could move from stock to scion apex [13, 94, 95]. Irrespective of FT protein, FT mRNA of *Arabidopsis* underwent long-distance movement from the stock to the scion apex [96]. The accumulation of FT proteins in the scion apex is partly derived from direct transport of stock FT proteins and others from the transport of FT mRNA and its translation.

Recent proteomic and metabolomic analyses suggest that grafting promotes the accumulation of proteins related to the synthesis of specific secondary metabolites. Only a few studies, however, have focused on whether these increasing proteins are derived from the translation of large amounts of their coding mRNAs or the movement from graft partners. On the basis of the investigations mentioned above, secondary metabolite-related proteins are potentially transported, or generated from the transported mRNA or both, and then lead to alterations in metabolites after grafting.

## Differential accumulation mechanism of one diterpene alkaloid

**Figure 4:**
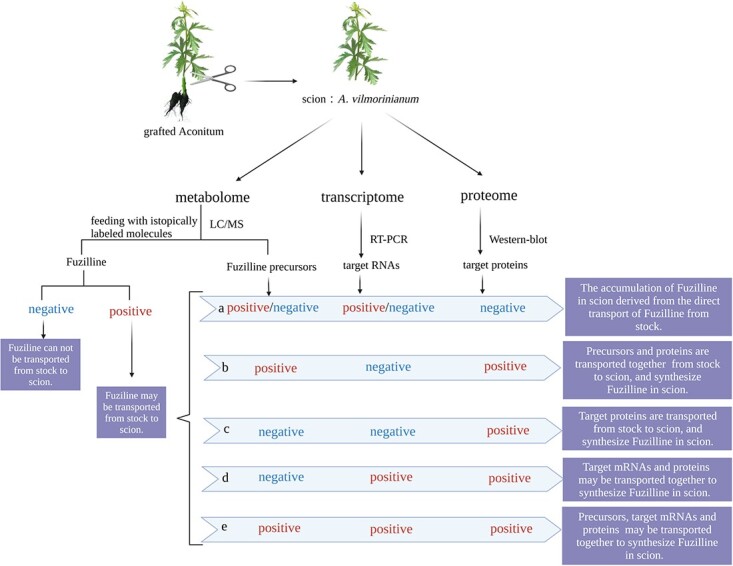
Flow chart for exploring the differential accumulation mechanism of fuziline.

As outlined above, grafting is an emerging technology for investigating the spatial transport and accumulation of the target metabolites. Based upon our previous work on diterpene alkaloids in *Aconitum* spp. [97–99], here we take fuziline, a typical diterpene alkaloid that accumulates to a high level in *Aconitum carmichaeli*, as an example to illustrate how to use grafting to reveal the transport and accumulation mechanisms of this secondary metabolite (Figs 3 and 4).

The transport directions of fuziline are determined by mutual grafting of *A. carmichaeli* and *A. vilmorinianum* and then detecting the presence of fuziline in the grafted *A. vilmorinianum* parts. Potential conclusions are given in Fig. 3a–d. As we mentioned above, differential accumulation of target products is not merely determined by the transport of the compound itself but is also due to the translocation of synthesis-related precursors, mRNAs or proteins. Here, we consider the possibility that fuziline is transported from stock to scion (Fig. 3b) to further elucidate how to elucidate the differential accumulation mechanism at multiple levels (Fig. 4).

Based on the appearance of fuziline via metabolome analysis, a series of experiments, e.g. transcriptomics and proteomics coupled with RT–PCR, western blotting, or other tools, are conducted to further confirm whether or not the presence of rootstock-specific fuziline in the scion is caused by the potential transfer of biosynthesis-related precursors (like *ent*-kaurene, a precursor of diterpene alkaloids), RNAs, or proteins. The target fuziline biosynthesis-related mRNAs and proteins are first picked out by comparative omics analysis of grafted materials and then are further verified by RT–PCR and western blot analysis (Fig. 4). The following are foreseeable consequences. (i) As long as the western blotting result is negative, the accumulated fuziline in the scion definitely derives from direct transport from stock, regardless of whether or not the precursors and mRNAs are transported (Fig. 4a). (ii) If RT–PCR is negative but western blotting and fuziline precursors are positive, this indicates that target precursors and proteins are transported together from stock to scion, and fuziline is synthesized in the scion (Fig. 4b). (iii) A positive western blotting result but negativity for RT–PCR and fuziline precursors suggests that the movement of fuziline-related proteins but not mRNA or precursors is the key factor in fuziline accumulation; the target proteins are transported to the scion and they use scion-derived precursors as substrates to synthesize fuziline (Fig. 4c). (iii/iv) Positive positivity for both RT–PCR and western blotting indicates that at least target mRNAs could be moved from stock to scion (Fig. 4d and e), but we cannot assert that the related proteins are transported from stock to scion as well, because of the existence of target proteins from the translation of coding mRNAs in the scion. Whether the target proteins are transported could be further determined by testing target proteins in the phloem sap of the scion.

**Figure 5:**
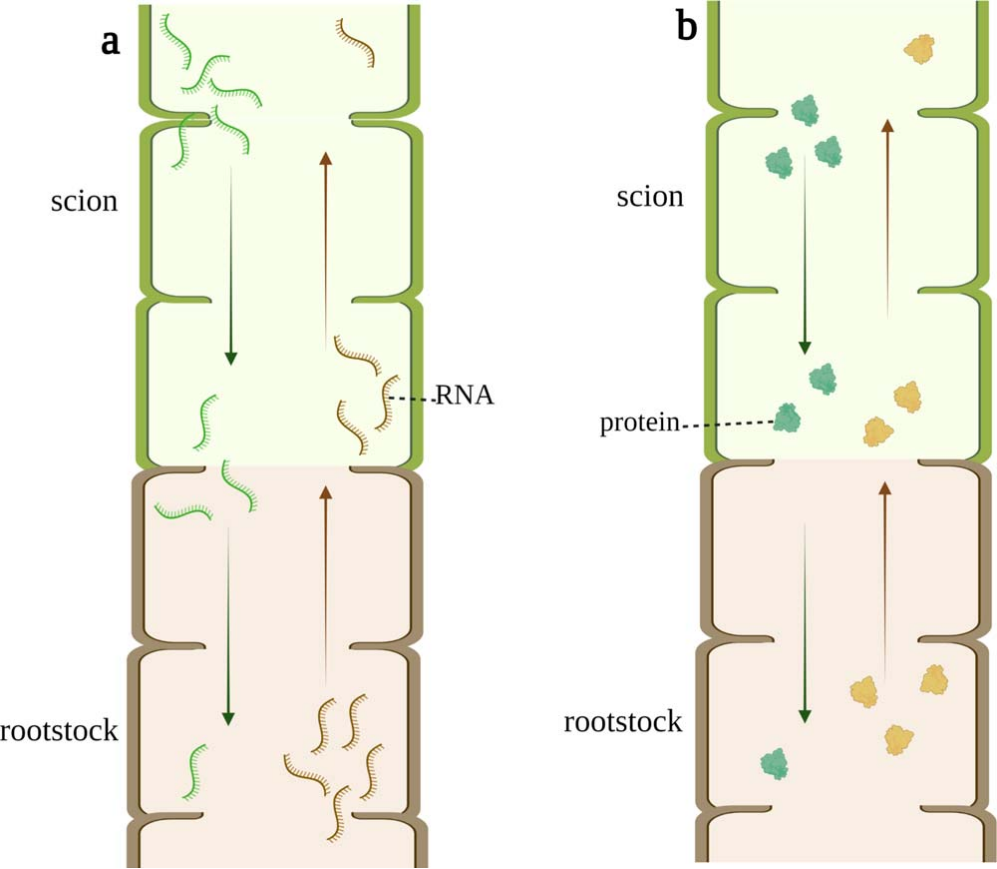
Degradation of RNA and protein in grafted plants. Green icons represent molecules from the scion and brown icons represent molecules from the rootstock. **a** RNA degradation during transport. **b** Protein degradation during transport.

The above process is outlined under an ideal experimental condition, but the feasibility of the proposed experimental design needs to be considered in the real situation. Generally, plants in the same family have more similar genetic backgrounds, which leads to the potential difficulty of designing specific RT primers or antibodies to identify the target mRNAs and proteins. Recently, it has become possible to create an increasing number of successful grafts of two plant families in distant phylogenetic relationship. For instance, *Nicotiana* species displayed great potential for interfamily grafting [100]. When 7 *Nicotiana* species were grafted to 84 species belonging to 42 families, perfect grafting combinations were found with 73 species in 38 families [100]. It is worth considering the use of *Nicotiana* species as a candidate grafting partner to study the transport and accumulation of specific metabolites of fuziline.

The case of fuziline we discuss above is mainly focused on the protein-encoding genes that are directly involved in the synthesis of fuziline. Grafting also leads to epigenetic modification and the transport of many transcriptional regulators, which participate indirectly in the regulation of their synthesis and accumulation. The mechanisms underlying these aspects require further exploration.

## Perspectives

Differential accumulation mechanisms for most secondary metabolites are still unknown. Many valuable secondary metabolites are derived from non-model plants, and the lack of mature genetic transformation systems or mutants limits further surveys of the differential accumulation mechanisms of these metabolites [101]. To a certain degree, reasoned selection of combinations of stock and scion, combined with integrated omics approaches and other tools, offers the possibility of solving this problem.

Furthermore, degradation of long-distance-transported mRNAs and proteins (Fig. 5) is general in planted plants, and is confirmed by mutant grafting of *Nicotiana benthamiana* [102] and *Jatropha curcas* [103]. The influence of the stock on the scion, therefore, will diminish as the scion grows. We cannot deny the presence of protein or RNA transport if they are not detected, due to their potential degradation during transfer.

## Author contributions

D.K.Z. and D.D. conducted the literature review and wrote the manuscript. The other authors provided comments and modified the manuscript. All the authors have reviewed and approved the final submission.

## Conflict of interest

The authors declare no competing interests.
